# Renal cyst masses (Bosniak category II–III) may be over evaluated by the Bosniak criteria based on MR findings

**DOI:** 10.1097/MD.0000000000009361

**Published:** 2017-12-22

**Authors:** Jianguo Zhong, Fang Cao, Xiaojun Guan, Junfa Chen, Zhongxiang Ding, Minming Zhang

**Affiliations:** a Department of Radiology, the Second Affiliated Hospital, Zhejiang University School of Medicine; b Department of Radiology, Zhejiang Provincial People’ Hospital, People’ Hospital of Hangzhou Medical College, Hangzhou, China.

**Keywords:** Bosniak classification, magnetic resonance imaging, pathology, renal cysts, tomography, x-ray computed

## Abstract

A classification system of renal cysts developed by Bosniak is based on computed tomography (CT) findings and has been applied to deal with the complex cystic renal masses. Magnetic resonance (MR) has excellent soft-tissue resolution, it has been used to further evaluate some complex renal lesions, especially those suspected of containing soft tissue components and hyperattenuating cystic lesions seen on CT. Compared with CT, MR images may find additional information, which may lead to inconsistent classification. However, at present, there is no consensus on the treatment of these inconsistent lesions. This study aimed to investigate the value of MR in the evaluation of renal cystic masses by using the Bosniak classification system and improve understanding of the MR features of renal cyst masses.

The present study retrospectively analyzed 35 renal cyst masses in 34 patients (10 men and 24 women with age from 20 to 65 years old, with an average of 49 ± 12.08), who underwent both MR and computed tomography (CT) examinations within 6 months (range from 1 to 135 days with an average of 11 ± 24.16 days). Twenty-four lesions (9 category III and 15 category IV on CT) received surgical treatment, 4 category IIF lesions on CT were upgraded to category III on MR, which were finally accepted operative resection. The remaining 7 lesions (category II–IIF on both CT and MR) were followed up for at least 3 years. For each lesion, size of both cyst and solid component, presence of calcification, number of septa, thickness of wall and septa, and appearance of enhancement were analyzed. Each lesion was categorized by using Bosniak criteria on CT and MR, respectively. The MR findings were compared with CT and pathology or follow-up results.

On MR, categories of the lesions were as follows: category IIF (n = 7), III (n = 12), IV (n = 16). On CT, categories of the lesions were as follows: II (n = 3), IIF (n = 8), III (n = 9), and IV (n = 15). Findings on MR and CT images were inconsistent in 8 (23%) lesions. Among them, 3 category II lesions on CT were classified as category IIF on MR images, 4 category IIF lesions on CT were upgraded to category III on MR, and 1 category III lesions to category IV. In these lesions, MR detected more increased wall/septa thickness (n = 8) and septa number (n = 3) than CT, resulting in an upgrade in classification. Based on the pathological results, 5 of category III (5/9, 56%) and all category IV (15/15, 100%) lesions on CT images were malignant. On MR, 4 of category III (4/12, 33%) and all category IV (16/16, 100%) lesions were malignant.

The renal cyst masses in some cases, especially category II to III lesions, may be over evaluated by the Bosniak criteria based on MR findings. It is necessary to combine MR features with CT findings in evaluation and management of these cases with renal cystic masses.

## 1 Introduction

Renal cyst masses can be classified into either simple cysts or complicated cystic lesions. The Bosniak classification criteria was introduced in 1986^[1]^ and has been used as an effective tool to evaluate renal cystic masses and guide clinical management.^[2–6]^ It was originally depended on the basis of computed tomography (CT) findings. Recently, it has been founded that the Bosniak criteria may be also useful on ultrasound (US) and magnetic resonance (MR) imaging. Several investigators compared the findings of CT and MR imaging according to the Bosniak classification criteria, and reached the conclusion that both imaging modalities got the similar results in evaluation of the majority of cystic renal masses.^[7–10]^ However, because of the superiority in soft tissue resolution, MR exhibited additional findings in some cases,^[8]^ such as more septum or irregular thickened walls/septum, or enhancement, which led to an upgraded cyst classification, especially in lesions classified II to III. Additionally, because of the inherent artifacts in magnetic resonance imaging, wall/septa in renal cystic masses may appear thicker than on CT, this may bring about disagreements, and lesions classified as II or IIF on CT images might be classified as IIF or III on MR.^[11]^ The significance of these category changes was still controversial and beg further investigation with better radiological–pathological correlative studies. Our study aimed to test the usefulness of MR findings in comparison with CT findings and pathological results in evaluating the renal cyst masses according to the Bosniak criteria.

## 2 Materials and methods

### 2.1 Study population

All the patients with the diagnosis of renal cyst masses on both CT and MR from January, 2012 to June, 2016 in the database of our hospital were retrospectively searched. Those patients received surgical treatment with pathological diagnosis or followed up for at least 3 years were included. This search resulted in a total of 63 patients with 64 masses. Among those subjects, patients with only CT (n = 19) or MR (n = 10) examination were excluded. Finally, 34 patients (10 males and 24 females) with 35 renal cyst masses were included in the study. The average age of the included subjects was 49 ± 12.08 years old (ranging from 20 to 65). The maximal gap between MR and CT scans was 135 days (range from 1 to 135 days with an average of 11 ± 24.16 days). Among the 34 patients, 20 patients were asymptomatic and diagnosed with renal cystic lesion in annual health check-up through an ultrasound examination. Nine patients presented with varying degrees of flank soreness, and the resting 5 patients suffered from hematuresis and/or discomfort during urination. This retrospective study was approved by Ethics Committee of the Second Affiliated Hospital, Zhejiang University School of Medicine with waiver of informed consent.

### 2.2 CT and MR imaging

All patients had undergone both enhanced CT and MR scans. CT scan was performed using a SOMATOM Definition AS 40 or 128 scanners (Siemens Healthcare, Erlangen, Germany). The scanning parameters were as follows: 120 kV, 180 to 210 mAs, 3 to 5 mm slice thickness reconstruction. After an unenhanced scan, 2 contrast-enhanced scans were obtained 30 and 90 seconds after the intravenous bolus (3.0 mL/s) administration of 1.0 mL/kg of an iodinated nonionic contrast agent, iopromide (Ultravist, 370 mgI/mL; Bayer Schering, Berlin, Germany). MR was performed on a GE Discovery MR 750 3.0 T scanner (GE Healthcare, Little Chalfont, UK) or a Siemens Magnetom Avanto 1.5 T MR scanner (Siemens Healthcare, Erlangen, Germany). The patients at least underwent transverse breath-hold T1-weighted dual imaging (T1WI) with a two-dimensional spoiled gradient-echo sequence, transverse respiratory gating T2-weighted MR imaging (T2WI) with a fast spin-echo sequence, and transverse nonenhanced and contrast-enhanced T1WI with a three-dimensional fat-suppressed interpolated spoiled gradient-echo sequence. The scan parameters of dual phase T1WI were as follows: repetition time/echo time (TR/TE), 180–200/2.33 (out phase) to 5.8 (in phase) millisecond (msec); flip angle, 60°; section thickness, 3 to 5 mm; intersection gap, 1 mm; matrix, 288 × 224; field of view (FOV), 35 to 40 cm. T2WI: TR/TE, 3000–6000/85–90 msec; flip angle, 120° to 180°; matrix, 320 × 256; section thickness, 5 to 8 mm; intersection gap, 1 to 1.5 mm; field of view, (256–304) × (320–380) mm. The dynamic T1WI was performed 15, 60, and 90 to 120 seconds after intravenous injection of 0.2 mL/kg of gadopentetate dimeglumine. The parameters were as follows: TR/TE 3.99/1.43 msec; flip angle, 12°; matrix, 224 × 256; FOV, (315–360) × (350–400); section thickness, 4 mm; interpolated section thickness, 2 mm.

### 2.3 Imaging analysis

CT and MR images were reviewed by 2 senior radiologists in consensus (with 22 and 25 years of experience in CT and MR diagnosis, respectively), who were blinded to the previous imaging reports and pathological results. For each lesion, size of both cyst and solid component, presence of calcification, number of septa, thickness of wall and septa, and presence of enhancement were analyzed. Based on above imaging findings, each lesion was categorized according to the Bosniak cyst classification^[1,12,13]^ on CT and MR images, respectively. To test the inter-rater agreement, we calculated the kappa value of CT and MRI evaluations, respectively, by using IBM SPSS Statistics version 19.0 (Armonk, NY).

The Bosniak classification criteria of renal cysts used 5 separate categories:

A Bosniak I cyst is a simple cyst which has a hairline-thin wall, without calcifications, septations, or enhancement.

A Bosniak II cyst is minimally complicated. It may show a few hairline-thin septa, with small or short segment calcification in the cyst wall/septa. Perceived (as opposed to measurable) enhancement is sometimes present. Homogeneously hyper-attenuating non-enhancing lesions with a diameter <3 cm are also included in this category.

Bosniak IIF cysts may demonstrate several thin internal septations and a minimal thickening of the wall with perceived enhancement. A few thick or nodular calcifications may be present. Totally intrarenal non-enhancing hyper-attenuating cysts with a diameter of ≥3 cm are included. A Bosniak IIF mass requires follow-up imaging to determine whether it is benign.

A Bosniak III mass may contain thick irregular wall or septa with measurable enhancement. Also, there may be thick and nodular calcifications.

Bosniak IV cysts have measurable enhancing nodular soft tissue components which are independent of the wall or septa. They are considered to be malignant until proven otherwise.

## 3 Results

Among 34 patients, 12 patients had a cystic mass on the right side, 21 on the left, and the other 1 was bilateral. Both the inter-rater correlation coefficients of CT and MRI evaluations showed an excellent agreement (kappa values are 0.919 for CT and 0.956 for MRI).

**Figure 1:**
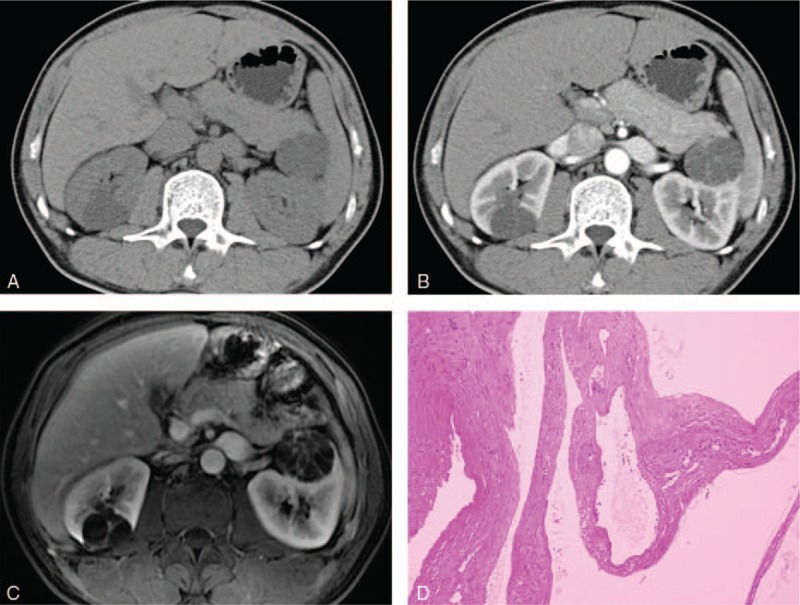
Images in a 45-year-old man with a cystic mass at the right and left kidney, respectively. (A, B) The right one at the axial contrast-enhanced CT scan demonstrated a minimally complex cystic mass that contains a few hairline-thin septa, suggesting a Bosniak category II cyst. The left one showed a complex cystic mass containing minimal thickened and enhancing wall and classified as a Bosniak category IIF cyst. (C) Gadolinium enhanced fat-suppressed T1-weighted MR image showed more septa than that on the CT scan, some of the septa are minimally thickened in the right lesion, and gross thickened septa depicted in the left lesion, caused the lesions to be upgraded to category IIF and III, respectively. (D) The left lesion was surgically removed and determined to be benign cyst. The right one underwent follow-up examinations for 36 months and showed no interval change. CT = computed tomography.

**Figure 2:**
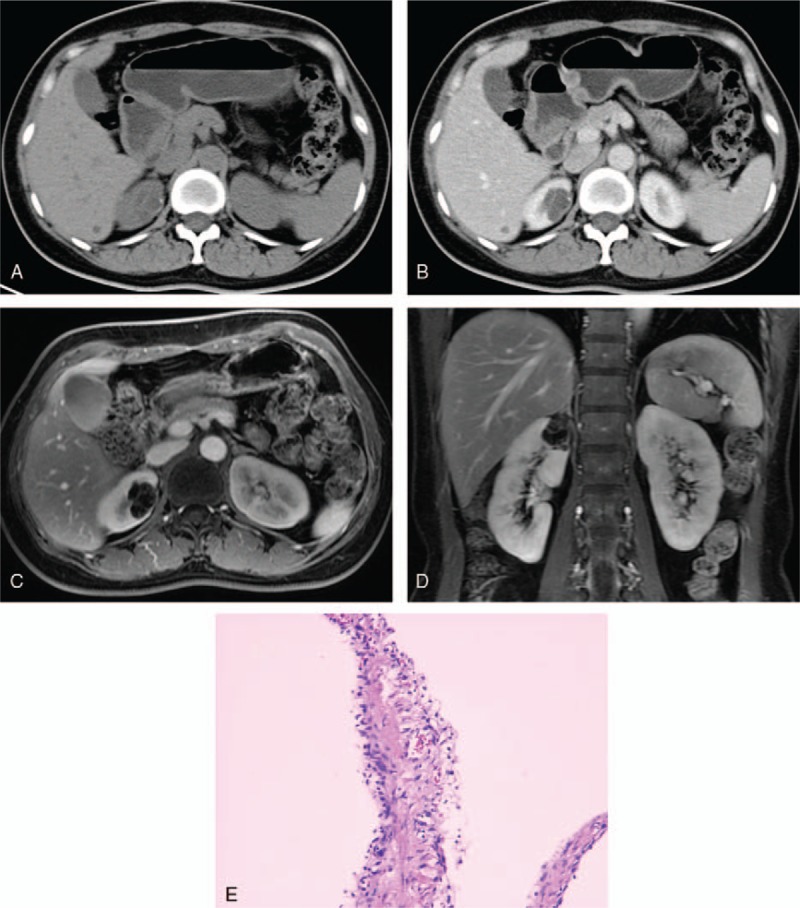
A 38-year-old woman with a cystic mass at the upper pole of the right kidney. (A, B) Axial plain and contrast-enhanced CT scans demonstrated a complex cystic mass with a few minimally thickened septa with perceived enhancement, and a short segment of slightly thickened calcification was depicted on the wall, was consistent with a category IIF cyst. (C, D) Axial and coronal gadolinium enhanced fat-suppressed T1-weighted MR image showed many septa, some of the septa were minimally thickened and others were confluent and had the gross thickening typical of category III lesions. (E) At surgery, a benign cyst was found. CT = computed tomography.

**Figure 3:**
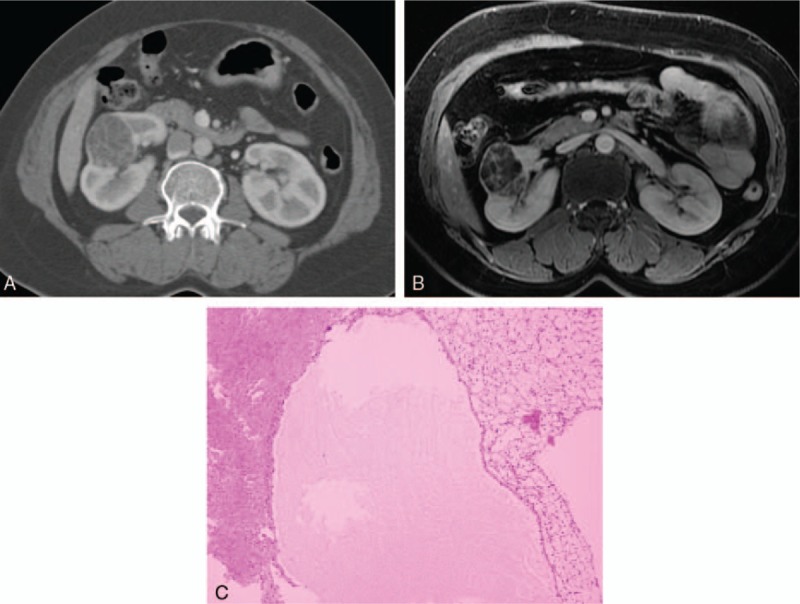
Images in a 45-year-old woman with a cystic mass in the right kidney. (A) Contrast-enhanced CT scan showed a complex cystic mass that contained a grossly thickened and enhancing septa, was consistent with a category III cyst. (B) Coronal Gadolinium-enhanced fat-suppressed T1-weighted MR image demonstrated more septa than are seen on the CT scan, and enhanced mural nodules were depicted on the wall/septa, caused the lesions to be upgraded to category IV. (C) This lesion was surgically removed and determined to be a renal cell carcinoma. CT = computed tomography.

Based on CT findings, categories of the lesions were as follows: II (n = 3), IIF (n = 8), III (n = 9), and IV (n = 15). On MR images, categories of the lesions were as follows: category IIF (n = 7), III (n = 12), IV (n = 16). Findings on MR and CT images were different in 8 (23%) lesions. Among them, 3 category II lesions on CT were classified as category IIF on MR images (Table, patients 1–3, Fig. 1), 4 category IIF lesions on CT were upgraded to category III on MR (Table 1, patients 4–7, Figs. 1 and 2) and 1 category III mass to category IV (Table, patients 8, Fig. 3).

**Table 1:**
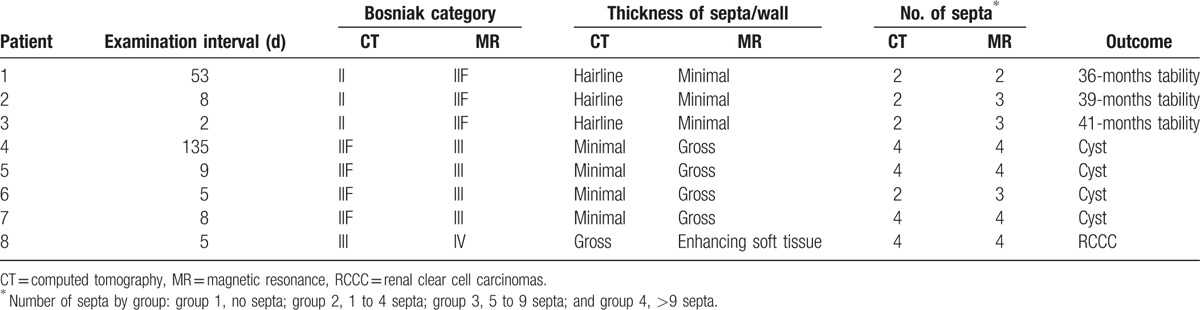
Comparison of discrepant CT and MR imaging findings in cystic renal masses.

Twenty-eight renal cysts received surgical treatment (9 category III and 15 category IV masses on CT findings, 4 category IIF masses on CT were upgraded to category III on MR) and pathological findings revealed 8 benign and 20 malignant lesions. Among the malignant lesions, 18 were renal clear cell carcinomas (RCCC), 1 were papillary renal cell carcinomas (PRCC), and 1 was spindle cell carcinoma. The Fuhrman nuclear grade of these lesions was 1 to 2. The proportion of malignancy increased with the Bosniak category rising. Based on the above pathological results, 5 category III (5/9, 56%) and all category IV (15/15, 100%) lesions on CT images were malignant, while 4 of category III (4/12, 33%) and all category IV (16/16, 100%) lesions were malignant on MR images. In addition, through pathological examination, there were 4 cysts with hemorrhage, 3 cysts with infection, and 1 cystic nephroma in the 8 benign lesions. They included 4 category IIF and 4 category III lesions on CT images, while all of the lesions classified as III on MRI (4 category IIF masses on CT were upgraded to category III on MR). The remaining 7 lesions classified as category II-IIF on CT were followed up for at least 3 years (range from 36 to 41 months with an average of 38.57 ± 1.98 months) and their lesions were unchanged. During the disease follow-up, they received at least 1 MR enhancement examination, among them, 3 category II lesions on CT were classified as category IIF on MR images, the other 4 cases have the same category (category IIF) on both CT and MR images.

## 4 Discussion

The Bosniak classification was developed for diagnosis and management of renal cyst masses firstly in 1986^[1]^ based on CT findings. It has been widely accepted and used by many radiologists and urologists, with general agreement between observers in most cases. Since MR has excellent soft-tissue resolution, it is more sensitive than CT to detect septa, solid component, and enhancement, which may result in the category upgrading and consequent more surgical probabilities. However, the outcomes of these category and management changes were still controversial and needed more radiological–pathological correlative researches.

In this study, we found that categories on MR were the same as that on CT in 27 of 35 lesions, while upgraded in 8 lesions. Three category II lesions on CT were classified into category IIF on MR images. They all presented more internal septa and smooth minimal thickened walls/septum, with perceived enhancement on MR images. One of them showed hemorrhage on MR and CT. Other 4 category IIF lesions on CT (with a smooth minimal thickened walls/septum, one presenting a patchy calcification on the wall), which all demonstrated grossly-thickened irregular walls and more septa with measurable enhancement on MR, were upgraded to category III. Two of them depicted hemorrhage on MR, while no obvious signs of hemorrhage were found on CT. The remaining 1 case was upgraded from category III on CT to category IV on MR because of enhanced mural nodule on MR image while that only showed irregular septa on CT.

The proportion of malignancy increased with the Bosniak category rising, as previous studies revealed. In our study, 28 renal cystic masses were treated surgically. Pathological findings revealed 8 benign and 20 malignant lesions. Twenty malignant renal cystic masses included 18 RCCCs, 1 PRCC, and 1 spindle cell carcinoma. There were 16 lesions on MR and 15 on CT were classified as category IV, all of which were malignancies. The only disagreement between CT and MR in category IV lesions was a renal clear cell carcinoma upgraded from category III on CT to category IV because of presenting enhanced mural nodule on MR images. Our results suggested that MR and CT showed a high degree of consistency in evaluating category IV cystic lesions, while MR was superior in detecting enhancement of solid components. Only 5 of the 20 malignancies didn’t contain mural nodules/independent soft-tissue components on CT and 4 on MR. Thus, mural nodules and/or independent soft-tissue components with enhancement on MR, as that on CT, may suggest malignant cystic lesions with high specificity. However, in the 8 benign lesions, there were 4 category inconsistent lesions (classified as Bosniak category IIF on CT but upgraded to Bosniak III on MR). Pathologically, they were all benign multi-locular cysts, including 1 with inflammatory granulation and hematoma, another accompanied by hemorrhage. The remaining 7 lesions with a Bosniak category of II-IIF on both CT and MR had no surgical indications. These patients underwent regular follow-up examinations for at least 3 years and showed no visible change in their lesions.

In 8 category inconsistent lesions, however, except 1 case of malignant tumor, 4 were confirmed as benign lesions, and 3 were considered to be stable lesions after 3 years of follow-up. Moreover, the frequency of malignancies in category III lesions on MR (4/12, 33%) was lower than that on CT in our study (5/9, 56%) and other former researches.^[12,13]^ The result of a meta-analysis including 9 CT researches pointed out a frequency of 65.3% in category III masses.^[14]^ Later, a study by Smith et al^[15]^ indicated that 54% of the category III cystic masses on CT were malignant. Above results showed that the Bosniak category on MR may cause upgrading and over-diagnosis in some cases, especially changing from II or IIF on CT to IIF or III on MR. This may result from the better demonstration of the presence of shin septa and solid components on MR images. To note, septa on MR may appear thicker than CT because of the inherent artifacts of MR imaging.^[11]^ Therefore, for a category III cystic mass on MR with grossly thickened wall/septa, if its CT category was IIF or below, management should be cautiously selected, weighing the risks and benefits of surgery in a particular patient based on all the clinical and radiological information.

There are some limitations in our study. Firstly, our study included a relatively small sample size, and further studies with more subjects and establishment of a new standard for evaluation of renal cystic mass on MR should be conducted. Secondly, the interval between the CT and MR examinations of patients varied from 1 to 135 days, for some cases, the secondary alterations to the disease progression between 2 scans would influence the evaluations.

In conclusion, MR and CT imaging has similar results in evaluation of renal cystic masses on the basis of the Bosniak classification in the majority of cases. However, more should be paid to for Bosniak IIF or III lesions based on MR, which contains thickened walls and more septa but no enhanced nodules/soft-tissue components. Complementary examinations including not only MR and CT findings but also complete clinical conditions, such as risk factors^[15]^ (coexisting category IV masses and history of renal malignancy) should be taken into consideration to improve the diagnostic accuracy.
